# Reduction of bilateral dislocation of TMJ and Rendu Osler Weber syndrome: case report and physiopathological model

**DOI:** 10.1093/jscr/rjy054

**Published:** 2018-03-29

**Authors:** L A Boccalatte, M G Nassif, M F Figari

**Affiliations:** 1 Head and Neck Surgery, Hospital Italiano de Buenos Aires, Buenos Aires, Argentina; 2 Maxillofacial Surgery, Hospital Italiano de Buenos Aires, Buenos Aires, Argentina

## Abstract

Temporomandibular joint dislocation (TMJ) is an infrequent clinical situation, representing 3% of all the human body’s dislocations. The etiological factors reported are associated to alterations typical of the joint or of the muscular-ligament apparatus, or to clinical conditions that may cause dislocation. We present the case of a 46-year-old patient with hereditary hemorrhagic telangiectasia with bilateral dislocation of the TMJ. There are several potential causes (antipsychotics, intubation, etc.) although the deposit of manganese in the basal ganglia that produce extrapyramidal symptoms could be the most consistent cause.

## INTRODUCTION

Temporomandibular joint dislocation (TMJ) is an infrequent clinical situation, representing 3% of all the human body’s dislocations [1]. The etiological factors reported are associated to alterations typical of the joint or of the muscular-ligament apparatus, or to clinical conditions that may cause dislocation, including laryngoscopy, endotracheal intubation, antipsychotic drugs or even systemic illnesses like Ehlers Danlos syndrome, orofacial dystonia or Marfan syndrome, among others [2].

It is not clear if there is an association between Rendu Osler Weber syndrome or hereditary hemorrhagic telangiectasia (HHT) and the dislocation of the TMJ.

We present the case of a 46-year-old patient with HHT with bilateral dislocation of the TMJ and the potential physiopathological theory of the event.

## CASE REPORT

The case of a 46-year-old male patient with a background of HHT and aortic valve replacement due to aortic insufficiency is presented. He denies having had psychiatrict treatment, consumed antipsychotic drugs, had recent molar extractions or a background of TMJ dislocation.

**Figure 1:**
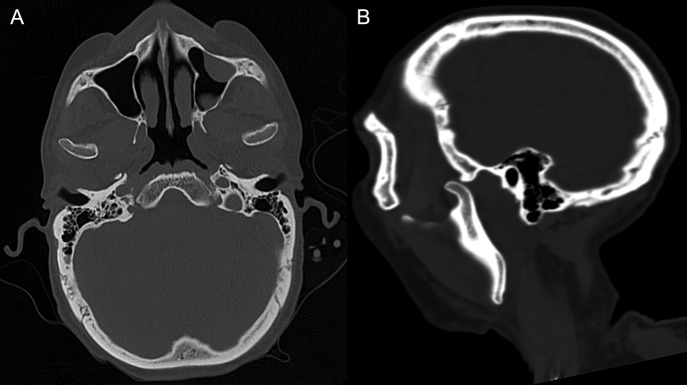
CT of the craniofacial skeleton without contrast showing the bilateral dislocation of mandibular condyles. (**A**) Shows as axial section and (**B**) sagittal section.

On the fifth day after a lung transplant, in the intensive therapy unit, the patient begins to experience symptoms of mandibular pain, trismus and extrapyramidalism symptoms in the form of oromandibular dystonia. In physical exam he presented signs of bilateral jaw subluxation with reuptake inhibition of the articular condyle–disc complex, contraction at the masticatory muscles level, fascies dolorosa, dystonia of the facial third with articular blocking and incapacity for bucal closure or lateral excursion and slight dysphagia. As background of the hospitalization, he had received, an hour before said event, 2.5 mg of haloperidol and 0.25 mg of risperidone (two doses) due to hyperactive delirium. It was decided to take a computed tomography (CT) of the craniofacial complex and brain without IV contrast (Fig. 1), where the following was seen: dislocation of both mandibular condyles with anterior position to the temporal’s glenoid cavity, without evidence of associated bone injuries.

Analgesia and muscular relaxation of the patient was carried out. The Nelaton reduction maneuver and later placing of Barton bandaging was done following the technique, using an antiseptic. Posterior clinical and radiological reduction is verified through CT, evidencing a normal condylar position. The patient progresses without further subluxations during his hospitalization.

## DISCUSSION

The dislocation of the TMJ occurs due to an imbalance between the muscular and structural component of the joint. The reported causes may be primary or secondary and are presented in Table 1. Following the etiologies presented, although it is feasible that orotracheal intubation and antipsychotic drugs may be the cause of the TMJ dislocation, these would not be the theories that are most connected with the case.

**Table 1:** Primary and secondary causes of temporomandibular dislocation.

Primary
Long-standing internal derangement.
Hiperlaxity of the articular disc and the capsular ligament.
Morphological changes of the glenoid fossa, zygomatic arch and squamotympanic fissure.
Spasm of the lateral pterygoid muscles.
Flattening or narrowing, decrease in the height of the articular eminence.
Secondary
Endotracheal intubation, laryngoscopy, trans oral fiber optic bronchoscopy.
Wide opening of the mouth while yawning, laughing, vomiting or seizures.
Dental treatments like third molar extractions or root canal treatments.
Antipsychotic medications.
Osteoarthritis, Ehlers–Danlos syndrome, Orofacial dystonia, Marfan syndrome.

The acute dystonia due to antipsychotic drugs varies in its impact depending on the antipsychotic used. In users of standard antipsychotics (like haloperidol), this oscillates between 2.3 and 60%, while users of atypical ones (like risperidone) oscilate between 2 and 3%. The likelihood of dystonia increases in adolescent patients, those with a recent history of psychosis, chronic use of antipsychotics, high doses and males [3]. In our patient, none of the risk factors were present, except for being male.

Regarding orotracheal intubation as a cause, works like those of Battistella *et al.* [4] have shown there are no statistically significant differences regarding the impact of TMJ disorders with muscular causes in patients subjected to intubation versus the control group. According to Martin *et al.* [5], the risk factors of TMJ dysfunction related with intubation are being female, interincisal distance, background of previous TMJ pain with or without headaches, and age. Our patient did not have any risk factors, the event occurred 6 days after instrumentation of the airway and the intubation during the transplant did not require excessive bucal aperture [6].

The HHT is characterized by spontaneous and recurrent epystaxis, telangiectasia in predetermined areas (oral cavity, nose, fingers and lips), visceral injuries (gastrointestinal, hepatic, pulmonary, cerebral and spinal), and a family history of HHT [7]. Recent research like that of Serra *et al.* [8] show that more than a third (4.6%) of patients with HHT have manganese deposits in the dorsal root ganglion. These deposits cause central level injuries that generate neuropsychological deterioration, including extrapyramidal symptoms like dystonia, being able to determine MTF dislocation just as occurred in our patient. Genetic studies [9] explain which deletions in 9q34.11 involve the genes ENG (endoglin), TOR1A (Early Onset Primary Dystonia), STXBP1 (Syntaxin Binding Protein 1) and SPTAN 1 are responsible for the multisystemic vascular dysplasia, early onset dystonia, epilepsy and intellectual impairment which shows the potential association between dystonia and HHT.

## CONCLUSION

Although more studies with greater scientific evidence need to be run to demonstrate an association between HHT and TMJ dislocation, this case study could be the starting point to investigate said association.

## References

[1] Lovely F, Copeland R Reduction eminoplasty for chronic recurrent luxation of the temporomandibular joint. J Can Dent Assoc 1981;47:179–84. 7013948

[2] Sharma NK, Singh AK, Pandey A, Verma V, Singh S Temporomandibular joint dislocation. Natl J Maxillofac Surg 2015;6:16–20. . PMC. 26668447 10.4103/0975-5950.168212 PMC4668726

[3] Burkhard PR Acute and subacute drug-induced movement disorders. Parkinsonism Relat Disorders 2014;20:S108–12. 10.1016/S1353-8020(13)70027-0 24262159

[4] Battistella CB, Ribeiro Machado F, Juliano Y, Guimarães AS, Tanaka CE, de Souza Garbim CT, et al Orotracheal intubation and temporomandibular disorder: a longitudinal controlled study. Rev Bras Anestesiol 2016;66:126–32. 25773451 10.1016/j.bjan.2014.06.009

[5] Martin MD, Wilson KJ, Ross BK, Souter K Intubation risk factors for temporomandibular joint/facial pain. Anesth Prog 2007;54:109–14. 17900209 10.2344/0003-3006(2007)54[109:IRFFTF]2.0.CO;2 PMC1993864

[6] Pillai S, Konia MR Unrecognized bilateral temporomandibular joint dislocation after general anesthesia with a delay in diagnosis and management: a case report. J Med Case Rep 2013;7:243. 24139071 10.1186/1752-1947-7-243 PMC4016029

[7] Shovlin CL, Guttmacher AE, Buscarini E, Faughnan ME, Hyland RH, Westermann CJ, et al Diagnostic criteria for hereditary hemorrhagic telangiectasia (Rendu-Osler-Weber syndrome). Am J Med Genet 2000;91:66e7. 10751092 10.1002/(sici)1096-8628(20000306)91:1<66::aid-ajmg12>3.0.co;2-p

[8] Serra MM, Besada CH, Cabana Cal A, Saenz A, Stefani CV, Bauso D, et al Central nervous system manganese induced lesions and clinical consequences in patients with hereditary hemorrhagic telangiectasia. Orphanet J Rare Dis 2017;12:92. 28521822 10.1186/s13023-017-0632-2 PMC5437640

[9] Campbell IM, Yatsenko SA, Hixson P, Reimschisel T, Thomas M, Wilson W, et al Novel 9q34.11 gene deletions encompassing combinations of four Mendelian disease genes: STXBP1, SPTAN1, ENG, and TOR1A. Genet Med 2012;14:868–76. 22722545 10.1038/gim.2012.65 PMC3713627

